# Pathways to Recovery: development and evaluation of a cognitive–behavioural therapy in-patient treatment programme for adults with anorexia nervosa

**DOI:** 10.1192/bjb.2017.30

**Published:** 2018-06

**Authors:** Andrea Brown, Richard Jenkinson, Julia Coakes, Annette Cockfield, Tish O'Brien, Louise Hall

**Affiliations:** 1 The Retreat, York, UK; 2 University of Sheffield, UK; 3 Insight Eating, Leeds, UK

**Keywords:** Anorexia Nervosa, Cognitive Behavioural Therapies, Comorbidity, Inpatient treatment, Outcome Studies

## Abstract

**Aims and method:**

A cognitive–behavioural therapy in-patient treatment model for adults with severe anorexia nervosa was developed and evaluated, and outcomes were compared with the previous treatment model and other published outcomes from similar settings.

**Results:**

This study showed the Pathways to Recovery outcomes were positive in terms of improvements in body mass index and psychopathology.

**Clinical implications:**

Adults with anorexia nervosa can achieve good outcomes despite longer illness duration and comorbidities.

**Declaration of interest:**

A.B., A.C. and L.H. work at The Retreat where the Pathways to Recovery were developed.

There is very limited guidance available regarding effective, evidence-based, in-patient treatment for those people with severe anorexia nervosa who cannot be managed safely due to high medical and/or psychosocial risk and/or who have not responded to treatment in out-patient services.^1^^,^^2^ Treasure *et al*^3^ have recently proposed that severe and enduring anorexia nervosa (SE-AN) may be defined by an illness duration of seven or more years and that interventions should be matched to illness ‘stage’. This proposed ‘staging model’ helpfully emphasises the importance of early intervention for people with recent onset anorexia nervosa. However, the suggestion of shifting focus away from recovery towards enhancing quality of life and harm minimisation for those with more enduring difficulties^4^^,^^5^ has the potential to fuel pessimism with regards to outcomes for this group of patients and the clinicians who work with them.

## Aims

This article aims to describe the development of a new treatment programme called Pathways to Recovery: an innovative cognitive–behavioural therapy (CBT)-based approach for the in-patient treatment of people with eating disorders, including those with significant chronicity and comorbidity.

The following questions are addressed:

• What is the outcome for patients treated by Pathways to Recovery?
• How do the outcomes of patients treated by Pathways to Recovery compare with the outcomes of patients treated by the previous approach that used body mass index (BMI) guidelines (i.e. treatment as usual (TAU))?
• How do the outcomes of patients treated by Pathways to Recovery compare with the outcomes in comparable in-patient settings?

## Method

### Development of the Pathways to Recovery model

The model was developed in a regional specialist eating disorder in-patient unit for women aged 18 and over with complex eating disorders and comorbidities including personality disorders, substance misuse and autistic spectrum disorders.

A CBT approach was adopted in response to evidence that CBT was the most effective treatment for bulimia nervosa^6^ and it had the potential to be effective trans-diagnostically with other types of eating disorders^7^ and a wide range of comorbidities. The collaborative CBT approach was not compatible with the pre-existing framework of using BMI to inform treatment and evaluate progress (i.e. the BMI guidelines).^8^ The Pathways to Recovery model was developed as a solution by a multidisciplinary group of clinicians with consultation with patients, their families/carers and the wider staff team. Pathways to Recovery incorporated key CBT principles and enabled the whole staff team to work collaboratively and coherently in an integrated and holistic way with each patient.

The Pathways to Recovery model was piloted and evaluated independently using qualitative methodology. Overall, Pathways to Recovery was found to be acceptable and a positive alternative to BMI guidelines.^9^

**Fig. 1:**
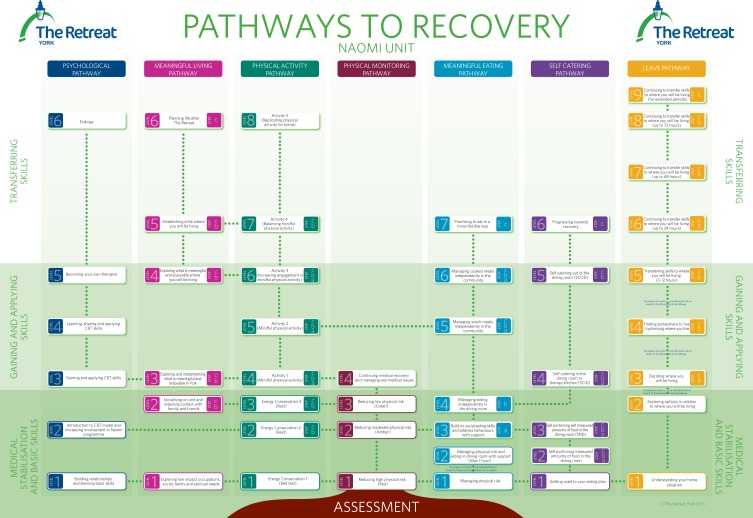
The Pathways to Recovery poster.

Pathways to Recovery broadly defines recovery as learning to live with eating-disordered thoughts without using eating-disordered behaviours. Emphasis is thus placed on behavioural change while developing a repertoire of CBT skills to manage difficult thoughts and feelings. The programme consists of seven parallel pathways: physical monitoring, psychological, physical activity, meaningful living, meaningful eating, self-catering and leave (Fig. 1). Progression up the pathways corresponds to the person's stage of recovery, moving from medical stabilisation through to gaining skills and concluding with the transferring skills stage. Progression is discussed collaboratively at weekly multidisciplinary team meetings and regular care programme approach reviews, which are run transparently with the patient present throughout the meeting. CBT principles, methods and techniques are central to each pathway; for example, the concepts of hierarchies, graded exposure, behavioural experiments, problem solving and goal setting are used. A guided discovery approach is encouraged for both staff and patients. In addition to the poster which delineates the pathways (Fig. 1), each patient receives a folder containing information about every step of each pathway. The information includes the aims of each pathway and defines what the person will be expected to work on at each stage. A complete set of supporting documentation is also provided, which includes: planning and evaluation forms for meals, self-catering, activity and leave. The emphasis is on learning from experiences, enabling the patient to take ownership of their recovery and to progress to higher levels of the pathways. As the person progresses, the level of independence increases and they receive less support from staff. This leads to increasing periods of home leave where the person can practice the skills they have learnt. The benefit of this is that patients and their families are able to gain confidence that they will be able to continue using their skills independently once they leave the programme.

### Participants and procedure

Clinical outcome data were reviewed from admission and discharge of 161 patients admitted consecutively from July 2008 (when routine data collection was initiated) until April 2015 (when the data were analysed). All patients provided written informed consent at the start of their admission for their data to be used anonymously for research and service evaluation purposes. This project did not require a research ethics committee review as it was a service evaluation project using outcome data routinely collected as a normal part of clinical practice on the unit.

Although data were collected for all patients admitted during this time frame, only data related to patients with a diagnosis of anorexia nervosa at admission were included (*n* = 123). Patients with additional comorbid diagnoses were included.

Further exclusion criteria were as follows: currently receiving treatment in the unit (*n* = 13), previous admission already included in the analysis (*n* = 15), present at the time the service model changed and thus received treatment under both models (*n* = 11) and did not have two or more sets of outcome data (*n* = 10). A total of 74 participants were therefore included. Of these participants, 25 were treated using the former BMI-guidelines approach (hereafter designated TAU) and 49 were treated using the Pathways to Recovery programme (designated Pathways to Recovery). Treatment completion was not routinely documented, so all patients were included in the data analysis providing they had at least one data set in addition to admission data.

### Measures

The key areas targeted by in-patient admission were symptoms of eating disorder, general psychological distress and weight restoration. To evaluate change in these areas, the following outcome measures commonly used in eating disorder research were selected for their clinical utility:

Eating Disorder Examination – Questionnaire (EDE-Q): A self-report measure assessing eating disorder symptoms over the previous 28 days.^10^ This instrument has good reliability and validity.^11^ High scores indicate greater eating disorder psychopathology.

Clinical Outcomes in Routine Evaluation Outcome Measure (CORE-OM): A widely used, generic, 34-item self-report measure of psychological distress. This instrument has good reliability and validity.^12^ High scores indicate greater psychological distress.

BMI: BMI (weight/height^2^) was recorded for each patient at admission and at discharge.

### Statistical methods

Independent samples *t*-tests were used to compare the demographic and clinical variables between the two groups at admission. To evaluate the efficacy of Pathways to Recovery, comparisons between admission and discharge scores were assessed in the Pathways to Recovery group using paired sample *t*-tests. Cohen's *d* effect sizes are presented to enable comparisons across outcomes. To put any detected changes into a clinical context, outcomes were also compared to evidence-based guidelines and cut-off points. Comparisons between the TAU and Pathways to Recovery groups were assessed using a 2 (group: TAU and Pathways to Recovery) × 2 (time: admission and discharge) mixed design ANOVA, with repeated measures on the time factor. Data were explored to check assumptions of multivariate analysis. All statistics were carried out using SPSS version 22.0 for Windows. Missing data were dealt with using pairwise deletion.

## Results

### Sample characteristics

The demographic characteristics and clinical features of the two groups are shown in Table 1. There were no significant differences between the two groups in terms of these variables. The mean age of the overall sample was 27 years (range 18–57 years). Of the participants, 67% had previously been admitted for at least one specialist eating disorder in-patient treatment. The mean duration of anorexia nervosa was more than 9 years (range 0–31 years). More than half (51.9%) of the participants had an illness duration of 7 years or more, thus meeting the criterion proposed by Treasure *et al*^3^ for SE-AN. On admission the participants were significantly underweight: 86% had a BMI lower than 16 kg/m^2^ and nearly one third (31%) had a BMI lower than 13 kg/m^2^. More than half (54%) of the participants had at least one comorbid diagnosis including borderline personality disorder, post-traumatic stress disorder, obsessive compulsive disorder, generalised anxiety disorder, alcohol and substance misuse, autistic spectrum disorder, depression and gender dysphoria.

**Table 1:** Demographic and clinical features of the sample by group

	TAU (*n* = 25)	Pathways to Recovery (*n* = 49)	Test	*P*-value
Age at admission (years)	27.64 (8.12)	26.65 (9.27)	*t* (72) = 0.45	*P* = 0.65
Ethnicity			*X*² (1) = 1.05	Fisher's *P* = 0.55
White (British)	25 (100%)	47 (96%)		
Black/African/Caribbean/Black British	0 (0%)	2 (4%)		
Age at onset of illness	16.07 (4.23)	17.44 (7.01)	*t* (52) = −0.71	*P* = 0.48
Length of illness at admission (years)	11.97 (10.19)	8.05 (6.91)	*t* (52) = 1.63	Levene's *P* = 0.11
Previous in-patient admissions	1.25 (0.97)	0.88 (0.95)	*t* (59) = 1.42	*p* = 0.16

Data are shown as mean (s.d.) unless otherwise indicated. TAU, treatment as usual.

### Question 1: What is the outcome for patients treated by Pathways to Recovery?

For participants who did not complete the programme, the last available set of outcome data was used as discharge data (Table 2). BMI data were available for all patients as this was recorded weekly. However, patients who dropped out within the first four weeks of their admission only completed one set of self-report questionnaires and were therefore excluded from the analysis.

**Table 2:** Clinical characteristics for Pathways to Recovery group

	Admission	Discharge
BMI	14.14 (1.65) *n* = 49	18.73 (2.3) *n* = 49 *P* < 0.001
CORE-OM	22.85 (8.22) *n* = 45	15.59 (8.30) *n* = 45 *P* < 0.001
EDE-Q	3.97 (1.59) *n* = 43	2.49 (1.42) *n* = 43 *P* < 0.001

Data are shown as mean (s.d.) unless otherwise indicated. BMI, body mass index; CORE-OM, Clinical Outcomes in Routine Evaluation Outcome Measure; EDE-Q, Eating Disorder Examination – Questionnaire.

There was a significant response to Pathways to Recovery in terms of improvements in BMI, general psychological distress and symptoms of eating disorder. At the point of discharge, the mean BMI had significantly increased from 14.1 (s.d. 1.7) to 18.7 (s.d. 2.3; *t* (48) = 13.32; *P* < 0.001; *d* = 2.78) for patients in the Pathways to Recovery group. A significant proportion of the group achieved a BMI ≥18.5 kg/m^2^ at discharge: 67% (33/49). The mean CORE-OM score decreased by 7.2 (*t* (44) = 4.50, *P* < 0.001, *d* = 0.88). On average, CORE-OM scores remained above the clinical cut-off point (10) at discharge; however, nearly one quarter of the patients (24.5%) were discharged with scores below the clinical cut-off point. The mean global EDE-Q score at discharge was also significantly lower compared to admission (*t* (42) = 5.04, *P* < 0.001, *d* = 0.93). The mean EDE-Q score had reduced to <1 s.d. above the community norm at discharge (mean = 1.55, s.d. = 1.21)^10^ compared to 2 s.d. above the community norm at admission. Almost one quarter of the patients (24.5%) had a global EDE-Q score of within 1 s.d. of the community norm at discharge, representing minimal eating disorder psychopathology.^13^

### Question 2: How do the outcomes of patients treated by Pathways to Recovery compare with the outcomes of patients treated by TAU?

There were no significant differences at admission between the two groups on any of the three measures used (Table 3).

**Table 3:** Clinical characteristics at admission and discharge, by group

		TAU	Pathways to Recovery
BMI	Admission	13.55 (1.89), *N* = 25	14.14 (1.65), *N* = 49
	Discharge	16.94 (2.32), *N* = 25	18.73 (2.3),^a^^,^^b^ *N* = 49
CORE-OM	Admission	20.87 (7.59), *N* = 21	22.85 (8.22), *N* = 45
	Discharge	17.09 (9.78), *N* = 21	15.59 (8.30),^a^ *N* = 45
EDE-Q	Admission	3.60 (1.68), *N* = 20	3.97 (1.59), *N* = 43
	Discharge	2.50 (1.85), *N* = 20	2.49 (1.42),^a^ *N* = 43

Data are shown as mean (s.d.) unless otherwise indicated. TAU, treatment as usual; BMI, body mass index; CORE-OM, Clinical Outcomes in Routine Evaluation Outcome Measure; EDE-Q, Eating Disorder Examination – Questionnaire.

a. *P* < 0.001 *v.* admission.

b. *P* < 0.01 *v.* discharge TAU.

**Fig. 2:**
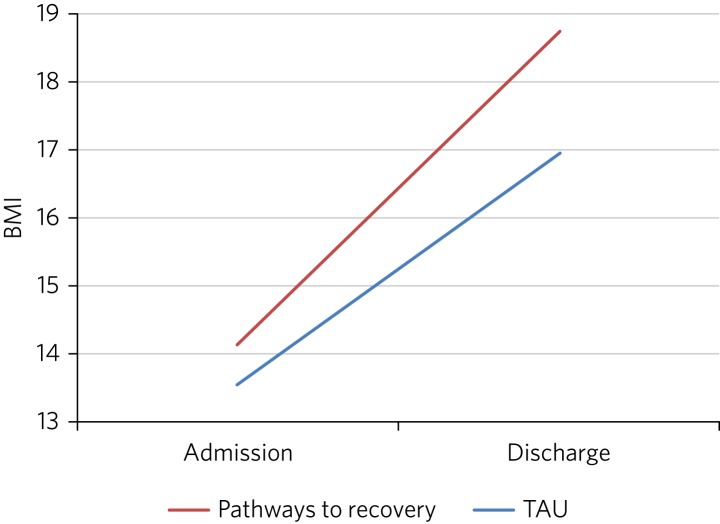
Effect of treatment on BMI.

A mixed ANOVA revealed a significant main effect of time (*F* (1, 72) = 221.67, *P* < 0.001) and group (*F* (1, 72) = 7.87, *P* = 0.01) on BMI. Both of these main effects were qualified by a significant interaction (*F* (1, 72) = 5.01, *P* = 0.03), which indicated that the change in BMI as a result of time was different between the two groups (Fig. 2).

The effect of treatment on BMI was thus greater in the Pathways to Recovery group than in the TAU group. To further explore this, simple-effect analyses were conducted. Independent *t*-tests revealed that although there was not a significant difference in BMI between the groups at admission (*t* (72) = −1.37, *P* = 0.17), the Pathways to Recovery group had a significantly greater mean BMI than the TAU group at discharge (*t* (72) = −3.16, *P* = 0.002).

A mixed ANOVA revealed a significant main effect of time (*F* (1, 64) = 16.89, *P* < 0.001) on CORE-OM. The main effect of group on CORE-OM was not significant (*F* (1, 64) = 0.02, *P* = 0.89), nor was there a significant interaction between time and group (*F* (1, 64) = 1.68, *P* = 0.20).

A mixed ANOVA revealed a significant main effect of time (*F* (1, 61) = 25.67, *P* < 0.001) on EDE-Q. The main effect of group on EDE-Q was not significant (*F* (1, 61) = 0.28, *P* = 0.60), nor was there a significant interaction between time and group (*F* (1, 61) = 0.57, *P* = 0.46).

The mean length of admission was 27.4 (s.d. = 14.55) weeks for TAU and 33.2 (s.d. = 17.47) weeks for Pathways to Recovery.

### Question 3: How do the outcomes of patients treated by Pathways to Recovery compare with the outcomes in similar in-patient settings?

The outcomes for Pathways to Recovery were compared to those reported by Dalle Grave *et al*,^13^ who describe findings from a CBT in-patient service in Italy, and Goddard *et al*,^14^ who describe outcomes from 12 adult in-patient services in the UK (Table 4).

**Table 4:** Pathways to Recovery outcomes compared to other in-patient settings

	Goddard *et al*^14^	Dalle Grave *et al*^13^	Pathways to Recovery
Mean length of admission (weeks)	26.4 (17.9)	Up to 20 weeks	33.2 (17.47)
Discharge BMI (kg/m^2^)	17.3 (2.1)	18.9 (1.5)	18.7 (2.3)
Those achieving BMI 18.5 (%)	22%^a^	86.1%^b^	67% (49%^a^)
Discharge EDE-Q	3.3 (1.6)	1.7 (1.0)^b^^,^^c^	2.5 (1.4)
Those achieving EDE-Q <1 s.d. above community mean (i.e. 1.74) (%)	Not known	51.4%^b^	24.5%

BMI, body mass index; EDE-Q, Eating Disorder Examination – Questionnaire.

a. Those achieving BMI >19 (%).

b. Only those who completed treatment rather than intent-to-treat sample.

c. EDE interview version rather than self-report.

#### Pathways to Recovery in comparison to Dalle Grave *et al*^13^

The mean discharge BMI in both settings was broadly similar. A greater proportion of the Italian patients left with a BMI in the healthy range (BMI 18.5–25 kg/m^2^) and met the criterion for minimal eating disorder psychopathology.

#### Pathways to Recovery in comparison to Goddard *et al*^14^

The Pathways to Recovery outcomes appear to be substantially better than the average outcomes reported by Goddard *et al*^14^ from 12 adult in-patient services in the UK: 49% of the Pathways to Recovery sample achieved a BMI greater than 19 kg/m^2^ at discharge compared to only 22% of the Goddard *et al*^14^ sample. The EDE-Q on admission for both groups was similar, and both achieved a statistically significant decrease in scores. However, the improvement in the Pathways to Recovery sample appears to be more clinically significant, achieving values closer to the mean taken from the general female population.

## Discussion

In addition to briefly describing the development of Pathways to Recovery, the aim of this paper was to evaluate the outcomes of this CBT-based approach for the in-patient treatment of people with severe and enduring anorexia nervosa. Although the programme is designed for people with any eating disorder diagnosis, the focus of this service evaluation was restricted to those with a diagnosis of anorexia nervosa. The people treated were complex, with almost one-third starting treatment with a BMI of less than 13 kg/m^2^ and more than half having one or more comorbid diagnoses. More than half the participants met the criterion of illness duration proposed by SE-AN.^3^ It is worth noting that the term SE-AN implies both a severe level of symptoms, including serious medical comorbidities as well a long duration of illness. Although people with shorter durations of illness may also experience physical comorbidities and other disabling features of anorexia nervosa, these symptoms become progressively more likely as time goes on.

The first key finding was that introducing Pathways to Recovery significantly enhanced weight restoration compared to TAU, with more than two-thirds of participants gaining enough weight to enter the World Health Organization's healthy BMI range. This is important since lower BMI at discharge has been found to be a predictor of relapse.^15^ Pathways to Recovery also led to significant improvements in general psychological distress and symptoms of eating disorder, with nearly one-quarter having minimal eating disorder psychopathology at discharge; however, these improvements were not significantly different from those achieved by TAU.

The outcomes for patients treated by Pathways to Recovery were broadly comparable to those produced by another CBT in-patient programme.^13^ The mean discharge BMI in both settings was similar. Although a greater proportion of the Italian participants left with a BMI in the healthy range (BMI 18.5–25 kg/m^2^) and met the criterion for minimal eating disorder psychopathology, this only represented the outcomes for those who had completed the programme; whereas the Pathways to Recovery data set includes those who did not complete the programme (except those who left within the first four weeks). Furthermore, the Dalle Grave *et al*^13^ sample included adolescents (29% were younger than 18), thus the mean age and median illness duration were less than the Pathways to Recovery sample. The Dalle Grave *et al*^13^ sample recorded depression (53.6%) and anxiety (20%) but did not report any other comorbidities. Depression and anxiety are recognised complications of starvation and may have been a feature of the anorexia nervosa rather than comorbidities *per se*. This could indicate a less complex cohort than the Pathways to Recovery sample. This indicates that Pathways to Recovery is effective for not only patients with SE-AN but also for those with other comorbidities. Such patient are often excluded from specialist eating disorder in-patient services due to the complexity of their presentations.

Another key finding was that Pathways to Recovery appears to produce better than average short-term outcomes for in-patients with anorexia nervosa when compared with similar adult in-patient settings in the UK.^14^ The length of illness in both groups was virtually identical and although the length of stay was longer for the Pathways to Recovery group, the outcomes demonstrated that instead of being treatment resistant, this group of patients can achieve positive outcomes in terms of weight restoration and improvements in psychopathology. This may be important for generating greater therapeutic optimism for clinicians working with patients on the more severe and enduring end of the spectrum, and thus give hope to these patients and their families. Improvements in both of these outcome measures are likely to result in sustained recovery post-discharge.

Treasure *et al*^3^ have suggested changing the focus of those with SE-AN ‘to improving quality of life and minimising discomfort rather than achieving optimal weight’. This approach runs the risk of clinicians and patients assuming that weight restoration is not possible or even acceptable. Furthermore, chronic low weight has many long-term health risks affecting all organ systems,^16^ many of which can potentially reduce life expectancy.

However, Calugi *et al*^17^ caution: ‘there are strong reasons to indicate that pessimism regarding the recovery prospects of patients with SE-AN may not be entirely justified and consequently steering away from a recovery model may be premature at this stage’. They go on to describe the 1 year follow-up treatment outcomes of their intensive enhanced CBT programme that reveal no significant differences between SE-AN and non-SE-AN patients in terms of BMI and EDE (global and brief symptom inventory) scores at the 12 month follow-up. The mean length of illness for their SE-AN group was 12 years.

Our experience is that offering hope in the form of a recovery-based program to patients with eating disorders – irrespective of length of illness, severity or complexity – is positively received. The uptake following assessment is high, with some patients requesting to be referred nationally.

As a service evaluation, this study inevitably has a number of limitations. The programme was devised, used and evaluated at The Retreat which could introduce potential bias. In addition, two of the authors (L.H. and A.B.) currently work in the service. The outcomes could be positively affected by the fact that the team was actively involved in the development of the programme. On the other hand, the adoption of this new way of working represented a significant cultural shift for the team. Unsurprisingly, a number of changes to the supporting materials and the processes were required, particularly over the first year or so. Any changes were made in collaboration with the participants and team. It is worth noting that no additional resources were used and the staffing levels remained consistent before and throughout the development and implementation of Pathways to Recovery. The only cost incurred was the printing of the materials and the graphics for the poster and folders.

In terms of the comparison between Pathways to Recovery and TAU (i.e. the in-patient programme before introduction of the new model), the patients were not randomised to the different treatment groups and thus there may have been variations between the two groups that could explain the differences in outcomes, despite there being no significant differences in the key demographic and clinical characteristics measured.

If patients had more than one admission, only their first admission was included in the data set (15 sets of data from 14 patients were excluded in total). It could be argued that using their most recent admission would have been more representative for evaluating the effectiveness of the programme in treating people with severe and enduring eating disorders, assuming that difficulties may be even further entrenched by the time people have had more than one admission. However, it may be the case that those who have had a previous admission do better in a subsequent admission as they are able to build on their experiences.

Furthermore, 10 patients were excluded due to having only one set of data. These patients who dropped out in the very early stage of their admission may represent a subset of even more complex cases, making the final sample somewhat self-selecting.

Although patients were contacted at 3, 6 and 12 months post-discharge and asked to complete self-report outcome measures, the uptake was poor and has therefore not been reported in this paper. We are currently investigating alternative methods to collect follow-up data including the use of digital technology. One of the differences between Pathways to Recovery and TAU is the emphasis on transference of skills and the development of increasingly high levels of independence and self-efficacy, which would predict that longer term outcomes are likely to be encouraging.

## About the authors

**Andrea Brown** is a consultant psychiatrist and psychotherapist at The Retreat, York. **Richard Jenkinson** is an assistant psychologist at the University of Sheffield. **Julia Coakes** is a consultant clinical psychologist and **Annette Cockfield** is an advanced dietetic practitioner at Insight Eating, Leeds. **Tish O'Brien**, now retired, was an occupational therapist and **Louise Hall** is a clinical psychologist at The Retreat, York. All authors work or have worked in the eating disorders service (The Naomi Unit) at The Retreat in York.
